# The ambiguities of social inclusion in mental health: learning from lived experience of serious mental illness in Ghana and the occupied Palestinian territory

**DOI:** 10.1007/s00127-023-02555-4

**Published:** 2023-08-28

**Authors:** Ursula M. Read, Hanna Kienzler, Suzan Mitwalli, Yoke Rabaia, Lionel Sakyi, Annabella Osei-Tutu

**Affiliations:** 1 University of Warwick Medical School, Coventry, UK; 2 Department of Global Health and Social Medicine, King’s College London, London, UK; 3 Institute of Community and Public Health, Birzeit University, Birzeit, Occupied Palestinian Territory; 4 Centre for Migration Studies, University of Ghana, Accra, Ghana; 5 Department of Psychology, University of Ghana, Accra, Ghana

**Keywords:** Human rights, Social inclusion, Mental health, Ghana, Palestine

## Abstract

**Purpose:**

Social inclusion of people living with serious mental illness is widely promoted. However, only limited consideration has been given to the meanings of social inclusion within different settings and the ways in which it is envisioned, negotiated, and practised. In this paper, we explore meanings and practises of social inclusion from the perspectives of people living with serious mental illness and their families in Ghana and Palestine and how this is shaped by differing political and socio-cultural contexts.

**Methods:**

This paper draws on comparative ethnographic research including participant observation and interviews with people living with mental illness and family members in Ghana and the occupied Palestinian territory. Data were triangulated and analysed using thematic analysis.

**Results:**

Participants described experiences of social inclusion and participation within communities, home and family life, friendships and social life, and work and livelihoods. This revealed how such experiences were variously shaped by differing political contexts and socio-cultural norms and expectations within the two settings. These in turn intersected with aspects such as gender roles, age, and socio-economic status. Aspirations for inclusion included greater awareness and understanding within society, accompanied by opportunities and support for meaningful inclusion at the political as well as community level.

**Conclusion:**

Findings point to the value of a contextual understanding of social inclusion, taking account of the impact of the wider socio-cultural, political, and economic environment. They also point to the need for an intersectoral approach, beyond communities and mental health services, to provide meaningful opportunities and support for social inclusion.

## Introduction

Social inclusion features prominently in the 2022 WHO World Mental Health Report which argues that supporting recovery from mental health conditions goes beyond formal health systems. Rather, people require support in maintaining independence, making social connections, participating in community activities, managing complex relationships, and accessing housing, work, and education [1]. “Full and effective participation and inclusion in society” is also a key principle of the UN Convention on the Rights of Persons with Disabilities (CRPD) [2]. The Convention draws on the social model of disability that “concentrates the disability experience not in individual deficiency, but in the socially constructed environment and the barriers that impede participation” [3].^1^ Hence, social inclusion, as conceptualised in the CRPD, requires support and services to enable people with disabilities, including psychosocial disabilities, to live and participate meaningfully in the community [7].

Social inclusion for people living with mental illness is advocated to reduce stigma and discrimination and remove social and structural barriers to full participation in society [8, 9]. Yet, there is a lack of clarity around what is meant by ‘social inclusion’ [10]. Concepts often focus on the physical environment and functional abilities, whilst excluding social and political dimensions [11]. Only limited consideration has been given to the meanings of social inclusion within different socio-cultural settings and the ways in which it is envisioned, negotiated, and practised in the day-to-day experience of people living with mental illness and their families [8, 12, 13]. Further, there is little research on the perspectives of people with psychosocial disabilities, particularly outside high-income settings, and in conflict and war-affected settings [13]. Our research begins to address this gap through ethnographic research with people living with serious mental illness and family caregivers in Ghana and the occupied Palestinian territory (oPt), both signatories to the UN CRPD. The two settings were chosen as they stand at differing points within the global mental health landscape. Ghana has become a focus for rights-based mental health activities, including legal and policy reform and community advocacy, with significant input from British and other international partners and donors [14–17]. In the oPt global investment into the mental health response has mainly focussed on emergency and humanitarian interventions following conflict-related violence, with fragmented systems-level development [18, 19]. In keeping with the WHO policy [1], both countries have made commitments to expanding community-based mental health care with the aim to support people living with mental health conditions in the community.

Researching social inclusion through a comparative ethnographic lens helps us to explore how the experiences of people living with mental health conditions and their families are variously shaped by political context and socio-cultural differences. It also provides insight into how these intersect with factors such as age, gender, socio-economic disadvantage and political conflict, shaping day-to-day experiences in domains such as neighbourhoods, family life, friendships, and work. Specifically, we focus on heterogenous meanings and practises of social inclusion for people with lived experience of mental illness and their families within these contexts and what, from their perspective, meaningful social inclusion could or should look like.

## Background

Social inclusion in mental health is used to refer to the extent to which people are able to meaningfully participate in all aspects of community life as equal citizens [1, 12]. Baumgartner and Burns argue that social inclusion consists of four key aspects: a sense of belonging and acceptance, active participation in the community, a sense of agency and capacity to choose, and opportunities for participation [10]. However, concepts of social inclusion contain several assumptions.

First, social inclusion assumes the community to be a desirable and identifiable social space within which to be included. Communities are often assumed to share a strong ethos of communitarianism and altruism which can prevent isolation and exclusion [8]. Indeed, studies show that people living with mental illness value meaningful engagement in activities, such as family life, parenting, household tasks, work, civil society, and religious and community events [20–22]. According to this research, meaningful participation has psychological, social and economic benefits, stimulating self-efficacy, reducing symptoms, and enabling the person to contribute towards the household and wider community [21, 22].

However, for people experiencing severe mental illness ‘inclusion’ in the community may also increase exposure to ridicule, abuse, and discrimination. For example, whilst the ‘extended family’ is often presumed to be the ideal environment to protect against such exposure and provide care and support [23, 24], it is seldom acknowledged that families can also be sites of conflict, tension, and strain. This may be more likely in the face of challenges arising from social, emotional and behavioural disturbance, costs of care, crowded and low-quality housing, and lack of state support [25–27]. Thus, social environments can be fraught and hostile rather than enabling and supportive [20].

Second, the drive for social inclusion assumes that the person must be able to behave according to the norms and values of the wider community [28]. Not participating appropriately may be judged as moral and social failure, particularly where interdependence and reciprocity are highly valued. Interventions often focus on rehabilitation or livelihood creation and target perceived individual deficits, rather than the structures which create and perpetuate inequalities and deepen social exclusion in the first place [29, 30].

A third assumption is that social inclusion operates in similar ways everywhere [12]. However, meanings and values associated with inclusion and the practises through which social inclusion is realised vary between contexts and individuals [8, 31], shaped by entrenched hierarchies along the lines of education, gender, ethnicity, and age. These can render social inclusion more achievable to some than to others. The ways in which people living with mental illness are excluded varies between contexts [10], as do the ways through which individuals, families, or other social groups imagine and enact ways to include and involve them [32].

Thus, whilst mental health policies and normative debates on social inclusion appear as straightforwardly beneficial, they can overlook the heterogeneity and complexity of situated experiences, needs, and opportunities and their intersection with socio-cultural, economic, and political contexts.

## Research settings

### Ghana

Ghana has a diverse population of nearly 32 m. Histories of the transatlantic slave trade and colonisation have left legacies of deep and lasting economic and social disadvantage. Economic growth has been accompanied by growing inequality [33]. Nationally nearly 30% of men aged 15–24 years are classed as unemployed and 17.4% of men aged 15–35 [34]. Two-thirds of the population are in insecure work [35].

In the last decade, there have been significant innovations in mental health policy and service provision in Ghana. This includes the 2012 Mental Health Act which aims to promote and protect the rights of people with mental illness [16] and an expansion in community mental health care [17, 36, 37]. Despite this, community mental health care remains primarily focussed on pharmaceutical treatment [17]. A few NGOs provide livelihood support and self-help groups [38], and more recently, people with lived experience of mental illness have become involved in advocacy and mutual support [17]. However, people living with serious mental illness in Ghana continue to experience stigma and exclusion [39], including difficulties in accessing employment and good quality, affordable mental health care [14, 40].

### Occupied Palestinian territory

The population of the oPt is estimated to be 5 million across the West Bank, Gaza Strip, and East Jerusalem [43]. The protracted Israel–Palestine conflict has exposed the Palestinian population to human rights abuses since the *Nakba* of 1948 and hindered institutional development [44, 45]. The Israeli military occupation starting in 1967 and the ensuing conflicts and Israeli settler attacks on neighbourhoods were characterised by intense violence. This has continued to the present day given the ongoing Israeli siege of the Gaza Strip and the illegal annexation of Jerusalem, combined with the Israeli-imposed movement restrictions, regular curfews and detentions, lethal force against civilians, including those with severe mental illness, land confiscations, and house demolitions [46–49].

This violence and intentional de-development [44] of infrastructure and essential services has been shown to severely impact on mental health in the Palestinian population [49–52]. In response, the Palestinian Authority established 16 Community Mental Health Centres [53, 54]. However, there is no mental health law and community support for people living with serious mental illness and their families is almost non-existent [51, 54]. People living with serious mental illness in oPt are often marginalised with lower levels of education and high levels of unemployment compared to the rest of the population [51, 55].

## Methods

The data presented form part of a 5-year ethnographic study (2017–2022) to understand what it means for persons with mental health conditions to live and participate in the community in Ghana and the West Bank of the oPt. The overall research programme consisted of participatory action research groups and ethnographic research with persons living with mental illness, family members, and key informants, including health providers, social workers, and community and religious leaders. In the following, we present findings based on participant observation, group discussions, and interviews with persons living with mental illness and family members.

### Data collection

Field sites were purposively selected to enable comparison across different rural/urban contexts within and between the oPt and Ghana. In the oPt, the study took place in villages and towns on the West Bank in the governorates of Ramallah, Qalqilya, Bethlehem, and Jericho. These were chosen as they span the north, centre, and south of the West Bank and reflect differing realities. In Ghana, research took place in the capital Accra and around Kintampo, Bono East region building on previous research in these locations [56, 57] (see Table 1 for details of the research sites).

**Table 1:** Research sites

Palestine	
Ramallah	The administrative centre where most resources are dedicated to mental health
Qalqilya	Surrounded by the Israeli separation wall and faces regular military and settler incursions
Bethlehem	Home to the only psychiatric hospital in the country
Jericho	An important agricultural area
Ghana	
Accra	The site of two of Ghana’s three dedicated psychiatric hospitals. As an expanding urban capital Accra has widening economic and social inequalities and rising costs affecting housing and food
Kintampo, Bono East region	A primarily agricultural market town surrounded by rural settlements. Kintampo is home to a health research centre hosting international research programmes and a training college for public health workers, including community mental health cadres [37]

Participant observation was used to gain insight into daily life for people living with serious mental illness and their families through observing household routines and interactions at workplaces, markets, cafes, places of worship, and mental health clinics. Observations, conversations, and group discussions were recorded in handwritten notes by the researchers which were later typed up and expanded.

Semi-structured interviews were carried out with adults aged over 18 who had been diagnosed with or showed behaviours consistent with serious mental illness, such as schizophrenia and bipolar disorder, and family members who lived with them or provided care (see Tables 2 and 3). Participants were identified through contacts within mental health services, NGOs, and advocacy organisations and word of mouth and purposefully recruited by combining criterion and snowball sampling. Interviews included questions about illness narratives, family life, social relationships, education, and employment. In the oPt, interviews were conducted in Arabic by SM and YR and by HK with the help of interpreters. In Ghana, interviews were conducted by LS and UMR in Twi and/or English. LS and another research assistant provided English interpretation. Interviews were recorded if permission was granted. If not, researchers took detailed notes.^2^

**Table 2:** Interview participants: people living with mental illness

	West Bank, oPt (*n* = 33)		GHANA (*n* = 15)	
	Female	Male	Female	Male
Gender	15	18	9	6
Age	Female	Male		
18–30	3	4	2	2
31–40	5	4	4	4
41–50	6	10		
51 +			3	
Marital status	Female	Male	Female	Male
Single	6	8	6	6
Married	3	7	1	
Divorced/separated	3	2	2	
Widowed	3	1		
Education	Female	Male	Female	Male
Primary	10	10	3	1
Secondary	2	5	4	2
Tertiary	2	1	2	3
Employment	Female	Male		
Employed/working	3	6	2	4
Unemployed	9	12	6	2
Retired			1

**Table 3:** Interview participants: family members

	West Bank, oPt (*n* = 26)		GHANA (*n* = 17)	
	Female	Male	Female	Male
Gender	23	3	13	4
Relation to person with mental illness				
Mother	10		7	
Father				1
Wife	9			
Husband				1
Daughter			2	
Son				1
Sister			4	
Brother	4			1
Other	3			
Age				
18–40	5		4	3
41–50	9		2	
51 +	10		7	1
Marital status	Female	Male	Female	Male
Single			1	1
Married			6	3
Divorced/separated			1	
Widowed			5	
Education	Female	Male	Female	Male
None			4	
Primary	13		5	
Secondary	7		1	2
Tertiary	6		3	
Employment				
Employed/working	9		8	2
Unemployed	6		3	2
Housewife	8			
Retired			1	1

### Analysis

Interviews were transcribed and translated into English where needed. Transcripts were triangulated with field notes and secondary data, such as policy documents and reports. All transcripts were read by at least two authors before coding combining deductive and inductive approaches [58, 59]. The data were analysed using thematic analysis [60]. Coded data were first categorised and links established between the categories. Through an interpretative, comparative process, we identified core themes and points of similarity and divergence within and between the two settings.

## Findings

### Experiences of inclusion and participation in different aspects of community life

In both settings, ‘the community’ was commonly described by participants as a valued physical and social space, reflecting ideals of collectivism and reciprocity. Palestinian participants emphasised equality, solidarity, and ‘Arab generosity’ towards the less fortunate. In Ghana, ‘community’ was associated with a particular location, such as a village or neighbourhood. During group discussions in Accra, people described community as ‘a place where they show love and support’ and where one felt a sense of ‘belongingness’. In group discussions in Kintampo, there was also emphasis on the community as a resource where ‘people come together to combine money to help one another’.

In practise, however, the community could be experienced as harmful or even dangerous. People living with mental illness described being exposed to hostility, humiliation, and, sometimes, abuse, including being mocked, ridiculed, and labelled as ‘mad’. People would stare at them, avoid them, talk about them behind their backs, and exclude them from common courtesies such as greetings. To fit in, many felt that they had to hide feelings and experiences that could single them out as being different. As a young Ghanaian woman in Accra explained: ‘belonging to a community first you have to conform and some of us do not want to conform' (ACNG09). In Palestine, inclusion and participation in the community were further shaped by the military occupation which induced insecurity, humiliation, and fear. This led people living with mental illness and their families to restrict their movements, avoiding certain places and people where they risked being targeted by the military.

### Home and family

Though the wider community could thus be experienced as hostile and excluding, everyday life was generally lived out within the nexus of familial relationships. The family home was the primary communal space where people received care and support. As indicated in Table 3, day-to-day family care was primarily the responsibility of women, specifically mothers. However, such care could be ‘complicated’ [61], operating in ways which, on the one hand, aimed to protect the person living with mental illness, but, on the other, prevented them from full and active social participation.

Families generally provided financial support and shelter, opportunities to take part in family life, and some protection from stigma and abuse. However, it was often difficult for people with severe mental illness to create their own families. As seen in Table 2, most participants living with severe mental illness were unmarried. A Palestinian man said, “If you take medication for your mental health they would look at you as crazy or as a stranger who they would avoid. They would look at me as a guy that they would never marry their daughter to” (ID27). Several women in both settings had been left by their husbands, leading to increased dependence on their natal family.

In Ghana and Palestine, events such as weddings and funerals provided an important space for interaction between the family and the wider community. However, the extent of involvement for people living with mental illness was dependent on factors including the perceived severity of symptoms, family attitudes, and normative expectations for participation. For example, a Palestinian man living with a mental health condition said, “of course, a wedding, an engagement for example in our family or even a funeral, it is very shameful not to go” (ID1). Other families excluded relatives living with mental health conditions from family events out of shame and concern that people might ridicule them. In Ghana, funerals form an important part of social life; however, some participants were prevented from attending when the body is publicly laid out for fear that they were too emotionally fragile or vulnerable to spiritual attack. One mother in Kintampo explained, “They say this sickness is a spiritual sickness, so when she goes the evil spirit can let something happen. So when someone dies and the person has such an illness we don’t let them go [to the funeral] until the corpse has been buried” (KTFM15).

Family care often took the shape of protection against the perceived hostility of the community, as well as against the shame of exposure for the person with mental illness and the family. A man in Kintampo described, for instance, how his mother had moved the family to a ‘remote place’ because of embarrassment about his behaviour: “Because of me, that’s why we came to this town […] you know Tema. Tema is a busy place. People would see you behaving strangely. She thought it wise to bring me to this place so that I would be out from the society” (KTPT08). Such protectiveness could be paternalistic and infantilising, denying the person agency and respect as an adult family member. Occasionally, this resulted in practises of confinement and restraint. In Palestine, for example, one mother complained about how her sons would lock their sister in her room when their friends came to visit due to their shame about her illness. In Ghana, some families used chains or shackles to physically restrain family members with mental illness who they feared might run away and get lost, use drugs, or harm others. Thus, though the family home was commonly experienced as a haven where people were supported, protected, and included, family care could also be paternalistic, discriminatory, and coercive.

### Friendships and social life

In both settings, participants reported a shrinking of their friendship networks following the onset of serious mental illness. Typically, this was due to struggles to interact with others as well as a lack of understanding from friends who sometimes blamed the person for their illness. In Ghana, beliefs that malign spiritual forces or perceived immoral behaviour can cause mental illness could increase social isolation. However, some participants formed new friendships within communities of peers who shared their mental health difficulties, for example Alcoholics/Narcotics Anonymous, WhatsApp groups, and, in Palestine, a community rehabilitation centre.

Participants also spoke about finding fellowship in their respective faith communities. Faith was valued as giving meaning and comfort and faith communities provided social support as well as access to networks and resources. A Palestinian man explained how attending the local mosque, conducting prayers, and observing Islamic practise gave him comfort. For many Christians in Ghana, the church provides an alternative family and sometimes a temporary home, particularly in times of acute mental health crisis. However, in both settings, religious communities could also be sites of moral judgement. In Ghana, people may also be subjected to harmful and demeaning practises, such as chaining and enforced fasting.

### Work and livelihoods

Participants in both countries highlighted work as an important way of achieving community inclusion as it provided them with some financial independence as well as a sense of meaning and purpose. However, most participants with longstanding mental illness were not working (see Table 2) or in irregular work and expressed frustration at their inability to support themselves and contribute to the household and wider society. Men, in particular, struggled to fulfil their expected role as family providers and could feel humiliated and inadequate by their dependence on others.

Women in both settings related differently to work and income generation compared to men. For instance, in Ghana, women are commonly expected to engage in income-generating activities such as ‘petty trading’—selling food, cosmetics, or baby clothes, to give a few examples—or helping with agricultural work. This was valued by participants as giving some financial independence, as one woman explained: “if I have money in my hands and I need to buy something, I can buy, I will not ask anyone. My brother pays for the electricity bill and everything, so I can’t go and ask for money from him” (KPTP13). Whilst almost none of the women interviewed in Palestine were employed, they participated in home-based work like cooking, cleaning, and taking care of children and ageing parents.

Despite being highly valued, in practise, work was often experienced as stressful and discriminatory. Participants in both settings described being refused employment if they disclosed their mental health status, being dismissed from work when they experienced a relapse and distrust or hostility from co-workers or customers. Others had felt compelled to resign, because they could not meet the demands of work and had no access to support or adjustments. For those in employment, long and rigid working hours, city commutes, and demands of professional work were felt to put people under ‘pressure’ and potentially trigger a relapse. Informal work could offer more flexibility, though such work was generally insecure and low-paid and required capital to establish.

## Aspirations for meaningful social inclusion

### The importance of acceptance and understanding

In both settings, meaningful social inclusion was envisioned to start with an increase in community awareness and understanding that everyone has the potential to be affected by mental illness. Providing support and combating stigma were therefore seen to be a concern for the whole of society. As a man living with recurrent mental illness in Kintampo put it, ‘Mental health, they say it could be anybody, it could even be you, it could be my brother, it could be everybody. So, you need not laugh at someone’ (KTPT08).

Greater understanding of mental illness was perceived to be the first step towards attitudinal change and solidarity, respect, and support for people with mental health conditions as members of society with equal rights. In Palestine, a young man living with mental illness explained: “People with mental illness should have rights and have obligations in return, and they should live in the community as normal people. There should be no discrimination and the community should be aware of mental illness” (ID37).

To counter discrimination and ensure full inclusion, participants living with mental illness in both settings explained the importance of being accepted for who they were, including their experiences of mental illness, rather than being put under pressure to hide their condition. Talking openly about feelings of distress was generally considered a sign of weakness or failure, particularly for men, and families tended to be unhappy about ‘showing dirty laundry in public’ to use a common phrase in Ghana. Nevertheless, people described the value of being able to talk honestly about their illness and share feelings of sadness, anxiety, or mania without being judged.

### Opportunities and support

Participants across both settings agreed that changing attitudes and awareness alone was not sufficient to translate into meaningful community inclusion and participation. According to some, achieving inclusion in areas such as education, work, housing, and social life required taking account of people’s differing needs and abilities and providing opportunities and support. For example, a retired teacher in Accra had lived with a psychotic illness all her adult life. She experienced unusual beliefs, heard voices, and had long periods when she was unable to work. However, despite these fluctuations in her mental health, her government employment meant that she continued to receive her salary and later her pension. She asserted her right to this support despite the fact that, as she put it: “I am different from everybody, I’m not everybody” (ACPT02).

Some participants expressed a desire for government investment in specialist services to support inclusion for people whose capacity to work was affected by their mental illness, such as rehabilitation centres, livelihood schemes, or supported employment. A Palestinian caregiver said: “I think that providing treatment centres where people with mental illness can participate economically would be helpful. When people with mental illness feel that they bring income, they would be better” (ID9). In Ghana, whilst some participants expressed a desire for government commitment and investment, this was largely focussed on providing reliable and affordable access to medication. There was an expectation that organisations such as NGOs, businesses and religious groups, should play a part in supporting livelihoods, for example by providing access to capital to start a business.

In sum, meaningful social inclusion for people with mental illness across the settings was perceived to require a holistic approach founded on empathy, tolerance, and acceptance to change public attitudes and understanding, alongside commitments from government, NGOs, and other agencies to provide consistent and equitable support and opportunities responsive to people’s changing needs.

## Discussion

Comparison within and across the two sites, Palestine and Ghana, allowed us to highlight key similarities and differences related to experiences of social inclusion due to differing histories, politics, and socio-cultural contexts, including aspects such as family structure, gender roles, and religion. Based on this, we argue that interventions and policy development for social inclusion must first understand the complexities and particularities related to lived experiences of community, family life, friendships, and livelihoods as well as the wider political and societal context in which they are embedded.

In terms of similarities, we found that in the absence of substantial government support, responsibility for social inclusion and participation of people living with serious mental illness was delegated to communities. Yet, experiences of living in the community were marked by ambivalence. Participants in both settings commonly articulated an ideal of the community as a place for support and belonging, providing familiar and safe spaces for participation. Simultaneously, they highlighted that communities can expose people living with mental illness to ridicule, hostility, and even violence.

Given such discrimination in the community, families provided both protection and support. Within the mental health literature, families have long been viewed as important assets to promote social inclusion [25]. Yet, in common with the other studies [26, 62], we show that whilst families do, indeed, play a major role in protecting and supporting people living with mental illness, they can also be sites of conflict, coercion, and exclusion, hindering recognition of their agency and rights [7]. Furthermore, for most families, their ability to provide adequate support was constrained by poverty and competing demands on their resources [25, 62]. Since women provided the majority of care, this could deepen existing gender inequalities [56].

Beyond this, our findings illustrate how expectations and opportunities for inclusion and participation were shaped by the wider context and specific socio-cultural norms [12, 20]. These played out in particular ways according to intersections with aspects such as gender, age, rural/urban contexts, and socio-economic status across the life course. In Ghana, for example, where it is not uncommon for women to be the major breadwinner in the household [63, 64], women as well as men emphasised opportunities for sustainable livelihoods as crucial to inclusion. In Palestine, on the other hand, fewer women were engaged in paid work compared to men but could meaningfully participate in everyday household activities. Women living with mental illness in this context generally had a smaller radius of social interaction beyond the household compared to men. This was further curtailed by movement restrictions and violence imposed by the Israeli military occupation which also affected men’s community participation beyond the household. A male participant described this coming together of mental illness, political violence, and community exclusion as a ‘triple whammy’ (ID20).

Across both settings, people living with serious mental illness thus faced significant social and structural barriers, meaning that social inclusion was only partially achieved, and often not on their own terms. In describing their visions for social inclusion, participants emphasised not only their desire for increased understanding of mental illness within wider society, but also for the provision of opportunities and supports which could make social inclusion possible, taking account of their particular needs and aspirations. Importantly, this included a desire for acceptance whilst recognising that there might be ongoing needs related to the illness, or the effects of treatment. This underscores that inclusion should not be predicated on treatment and rehabilitation to remove symptoms but, as argued within the capabilities approach, requires positive action to provide opportunities and support for people to live lives they value as equal and respected members of society, regardless of disability [7, 65]. As we have shown, what makes up this ‘good life’ is shaped by context, including gendered roles and aspirations [66–68].

Participants also pointed to ways in which social inclusion could be meaningfully enabled within different contexts. Joining with others who shared experiences of living with mental illness could offer recognition, empathy, and hope, as well as opportunities to amplify their voices in advocacy and activism to promote their rights [69, 70]. In Palestine, as yet such opportunities have been limited, though histories of political organisation could inform attempts to foster solidarity and claim rights. In Ghana, international funding and NGO programmes have given impetus to efforts to mobilise people with lived experience for mutual support and advocacy [15, 38]. However, such programmes tend to be unevenly distributed and activities may not be sustained once projects and funding come to an end. Furthermore, amplifying the voices of people with lived experience can have limited impact unless this is accompanied by actions to create ‘receptive social environments’ for transformative change [71]. This includes forging alliances to reach those who hold access to resources and opportunities, as well as strengthening moral arguments for equity and inclusion, for example through aligning with anti-colonial and feminist struggles for rights and recognition [70–72].

To date, there has been limited research into how such this can be achieved in low resource settings. A practise-based review in India, Nepal, and Afghanistan stressed the importance of strengthening the voices of people with psychosocial disability; understanding the socio-cultural context; recognising the role of the family; promoting grassroots collective action, strengthening public health systems and policy development, and promoting linkages between people with lived experience and affected communities to inform organisational actions [12]. Increasingly, there are calls for such intersectoral ‘whole of society’ approaches to mental health [1, 13, 73]. Kienzler and colleagues argue that “meaningful and diverse support mechanisms, and monitoring and evaluation of such support must be participatory involving various stakeholders, including persons with psychosocial disabilities and their families, the general public, employers, social workers, health providers, and policy makers” [13] (p. 17). This echoes aspirations voiced by participants in this study, who desired support and resources to enable families to provide care as well as safe social environments where they could feel welcome, supported and included. Whilst in both contexts, they highlighted a need for more accessible and affordable mental health services, they also demanded action to ensure their right to participate in areas such as education, work, and housing [1, 13, 40]. However, as some participants described, this can only be achieved if there is political will and investment to ensure that policy change is accompanied by action.

## Conclusion

Our findings provide an insight into the experiences of people living with serious mental illness in Palestine and Ghana and the ways in which intersections of family life, socio-cultural expectations, and the political and economic environment can come together to impact on possibilities for social inclusion. This extends the social of inclusion beyond the family and immediate community, recognising that participation in societies on an equal basis with others, as mandated in the CRPD, requires action and alliances at various levels from the local to the global [70] to make this a reality rather than remain an aspiration.

## Footnotes

1 Despite the deployment of the social model of disability in the UN CRPD, there are debates around the application of disability discourse to mental illness. Arguments include that casting mental illness as disability pathologizes experience and does not fit with the fluctuating course experienced by many. [4, 5]. Nonetheless, service user organisations were involved in the development of the CRPD and have since used the convention to argue for their rights, particularly in relation to freedom from coercive treatment (Article 12) [6].

2 One participant in Ghana and 3 in Palestine chose not to be audio-recorded.
